# 2,6-Bis(4-methoxy­phen­yl)-4-phenyl­pyridine

**DOI:** 10.1107/S1600536809051277

**Published:** 2009-12-04

**Authors:** Xiaoning Dong

**Affiliations:** a College of Life Sciences and Chemisrey, Tianshui Normal University, Tianshui, 741000, People’s Republic of China

## Abstract

In the title compound, C_25_H_21_NO_2_, which was synthesized by the condensation of 2,6-bis­(4-methoxy­phen­yl)-4-phenyl­pyridinium tetra­fluoro­borate with ammonia under microwave irradiation and solvent-free conditions, the angles between the central pyridine ring and the three benzene rings are 22.3 (2), 35.3 (2) and 19.8 (2)°. In the crystal, inter­molecular C—H⋯π hydrogen-bond inter­actions link the mol­ecules.

## Related literature

For the biological properties of pyridines, see Keys & Hamilton (1987 ▶); Chen *et al.*(1995 ▶). For related structures, see: Ondráček *et al.* (1994 ▶).
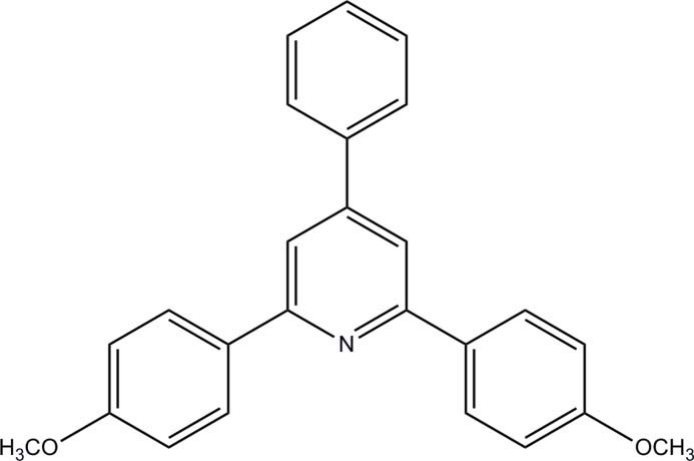


## Experimental

### 

#### Crystal data

C_25_H_21_NO_2_
*M* *_r_* = 367.43
Monoclinic, 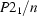

*a* = 6.379 (3) Å
*b* = 15.538 (8) Å
*c* = 20.51 (1) Å
β = 94.281 (7)°
*V* = 2027.3 (17) Å^3^
*Z* = 4
Mo *K*α radiation
μ = 0.08 mm^−1^
*T* = 298 K
0.41 × 0.18 × 0.08 mm

#### Data collection

Bruker SMART APEX CCD area-detector diffractometer
Absorption correction: multi-scan (*SADABS*; Sheldrick, 1996 ▶) *T*_min_ = 0.970, *T*_max_ = 0.994
10098 measured reflections
3566 independent reflections
1522 reflections with *I* > 2σ(*I*)
*R*_int_ = 0.109

#### Refinement

*R*[*F* ^2^ > 2σ(*F* ^2^)] = 0.099
*wR*(*F* ^2^) = 0.191
*S* = 1.04
3566 reflections
255 parameters
H-atom parameters constrained
Δρ_max_ = 0.17 e Å^−3^
Δρ_min_ = −0.16 e Å^−3^

### 

Data collection: *SMART* (Siemens, 1996 ▶); cell refinement: *SAINT* (Siemens, 1996 ▶); data reduction: *SAINT*; program(s) used to solve structure: *SHELXS97* (Sheldrick, 2008 ▶); program(s) used to refine structure: *SHELXL97* (Sheldrick, 2008 ▶); molecular graphics: *SHELXTL* (Sheldrick, 2008 ▶); software used to prepare material for publication: *SHELXTL*.

## Supplementary Material

Crystal structure: contains datablocks I, global. DOI: 10.1107/S1600536809051277/bq2177sup1.cif

Structure factors: contains datablocks I. DOI: 10.1107/S1600536809051277/bq2177Isup2.hkl

Additional supplementary materials:  crystallographic information; 3D view; checkCIF report

## Figures and Tables

**Table 1:** Hydrogen-bond geometry (Å, °)

*D*—H⋯*A*	*D*—H	H⋯*A*	*D*⋯*A*	*D*—H⋯*A*
C25—H25*C*⋯*Cg*1^i^	0.96	2.82	3.605	140

Symmetry code: (i) 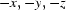. *Cg*1 is the centroid of the C19–C24 ring.

**Fig. 1.**
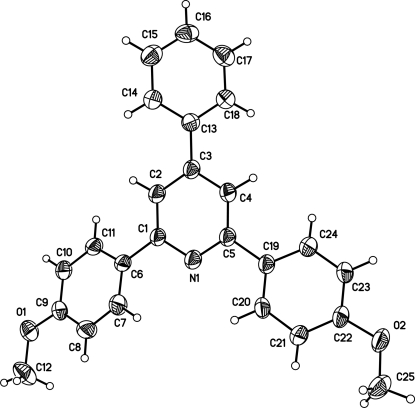
ORTEP drawing of the title complex with atomic numbering scheme and thermal ellipsoids at 30% probability level.

**Fig. 2.**
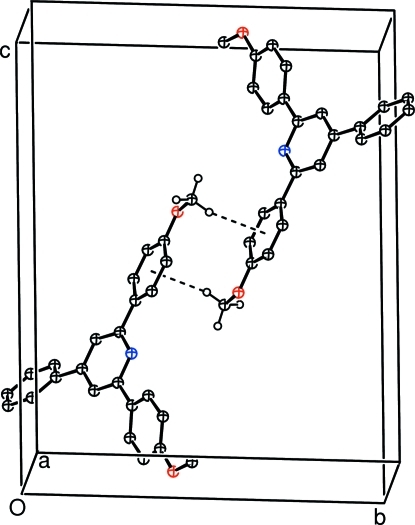
The one-dimensional chain structure was built by the C—H···π H-bond interactions (dashed lines).

## supplementary crystallographic information

### Comment

It was well known that pyridine ring systems have represented an important class of compounds not only for their theoretical interest but also because they displayed strong biological activity (Keys, *et al.*, 1987). Moreover, pyridine derivatives have remarkable versatility in synthetic organic chemistry as intermediates in the preparation of nature products and as ligands recently used in asymmetric synthesis (Chen, *et al.*, 1995).

In this paper, we present a new crystal, 2,6-bis(4'-methoxyphenyl)-4-phenylpyridine, (I), which was synthesized using benzaldehyde and 4-methoxylacetophenone as starting material.

In (I) (Fig. 1), the bond lengths and angles are normal and comparable to those observed in reported the compound (Ondráček *et al.*, 1994). The angles between the center pyridine ring and benzene rings (c6 - *c*11), (*c*13 - *c*18) and (*c*19 - *c*24) are 22.28 (19)°, 35.29 (18)° and 19.81 (20)°, respectively, which show the pyridine ring and three benzene rings aren't coplanar. Moreover, the crystal supramolecular structure was built from the connections of C—H···π hydrogen bonding interactions, as shown in Table 1 and Fig. 2.

### Experimental

Benzaldehyde (0.3 mmol) and 4-methoxylacetophenone (0.6 mmol), ammonia (1.0 mmol) under boron trifluoride ether (0.1 mmol) catalyzed, the mixture were mixed in 50 ml flash. After eradiating 3 min at 375 W, the mixture then cooled slowly to room temperature affording the title compound, then recrystallized from ethanol, affording the title compound as a colorless crystalline solid. Elemental analysis: calculated for C_25_H_21_NO_2_: C 81.72, H 5.76, N 3.81%; found: C 81.68, H 5.75, N 3.72%.

### Refinement

All H atoms were positioned geometrically, with C—H=0.93- 0.96 Å, and refined as riding, with *U*_iso_(H)=1.2–1.5*U*_eq_(C).

### Crystal data

**Table:**

C_25_H_21_NO_2_	*F*(000) = 776
*M**_r_* = 367.43	*D*_x_ = 1.204 Mg m^−^^3^
Monoclinic, *P*2_1_/*n*	Mo *K*α radiation, λ = 0.71073 Å
Hall symbol: -P 2yn	Cell parameters from 964 reflections
*a* = 6.379 (3) Å	θ = 2.6–25.1°
*b* = 15.538 (8) Å	µ = 0.08 mm^−^^1^
*c* = 20.51 (1) Å	*T* = 298 K
β = 94.281 (7)°	Needle, colourless
*V* = 2027.3 (17) Å^3^	0.41 × 0.18 × 0.08 mm
*Z* = 4	

### Data collection

**Table:**

Bruker SMART APEX CCD area-detector diffractometer	3566 independent reflections
Radiation source: fine-focus sealed tube	1522 reflections with *I* > 2σ(*I*)
graphite	*R*_int_ = 0.109
phi and ω scans	θ_max_ = 25.0°, θ_min_ = 1.7°
Absorption correction: multi-scan (*SADABS*; Sheldrick, 1996)	*h* = −7→7
*T*_min_ = 0.970, *T*_max_ = 0.994	*k* = −18→16
10098 measured reflections	*l* = −24→22

### Refinement

**Table:**

Refinement on *F*^2^	Primary atom site location: structure-invariant direct methods
Least-squares matrix: full	Secondary atom site location: difference Fourier map
*R*[*F*^2^ > 2σ(*F*^2^)] = 0.099	Hydrogen site location: inferred from neighbouring sites
*wR*(*F*^2^) = 0.191	H-atom parameters constrained
*S* = 1.04	*w* = 1/[σ^2^(*F*_o_^2^) + (0.0521*P*)^2^ + 0.3542*P*] where *P* = (*F*_o_^2^ + 2*F*_c_^2^)/3
3566 reflections	(Δ/σ)_max_ < 0.001
255 parameters	Δρ_max_ = 0.17 e Å^−^^3^
0 restraints	Δρ_min_ = −0.16 e Å^−^^3^

### Special details

**Table:**

Geometry. All e.s.d.'s (except the e.s.d. in the dihedral angle between two l.s. planes) are estimated using the full covariance matrix. The cell e.s.d.'s are taken into account individually in the estimation of e.s.d.'s in distances, angles and torsion angles; correlations between e.s.d.'s in cell parameters are only used when they are defined by crystal symmetry. An approximate (isotropic) treatment of cell e.s.d.'s is used for estimating e.s.d.'s involving l.s. planes.
Refinement. Refinement of *F*^2^ against ALL reflections. The weighted *R*-factor *wR* and goodness of fit *S* are based on *F*^2^, conventional *R*-factors *R* are based on *F*, with *F* set to zero for negative *F*^2^. The threshold expression of *F*^2^ > σ(*F*^2^) is used only for calculating *R*-factors(gt) *etc*. and is not relevant to the choice of reflections for refinement. *R*-factors based on *F*^2^ are statistically about twice as large as those based on *F*, and *R*- factors based on ALL data will be even larger.

### Fractional atomic coordinates and isotropic or equivalent isotropic displacement parameters (Å^2^)

**Table:**

	*x*	*y*	*z*	*U*_iso_*/*U*_eq_	
N1	0.2077 (6)	0.7947 (2)	0.20968 (17)	0.0595 (10)	
O1	−0.0669 (6)	0.8942 (2)	0.49408 (14)	0.0804 (11)	
O2	−0.0509 (6)	0.9151 (2)	−0.08307 (14)	0.0762 (10)	
C1	0.2860 (7)	0.7601 (3)	0.2683 (2)	0.0539 (12)	
C2	0.4486 (7)	0.7015 (3)	0.2720 (2)	0.0608 (13)	
H2	0.4984	0.6806	0.3127	0.073*	
C3	0.5412 (7)	0.6725 (3)	0.2161 (2)	0.0569 (12)	
C4	0.4592 (7)	0.7086 (3)	0.1567 (2)	0.0602 (13)	
H4	0.5156	0.6927	0.1180	0.072*	
C5	0.2955 (7)	0.7675 (3)	0.1551 (2)	0.0550 (12)	
C6	0.1864 (7)	0.7938 (3)	0.3266 (2)	0.0542 (12)	
C7	−0.0101 (8)	0.8304 (3)	0.3224 (2)	0.0724 (15)	
H7	−0.0854	0.8318	0.2817	0.087*	
C8	−0.1032 (8)	0.8657 (3)	0.3762 (2)	0.0759 (16)	
H8	−0.2356	0.8909	0.3712	0.091*	
C9	0.0074 (8)	0.8620 (3)	0.4375 (2)	0.0613 (13)	
C10	0.2024 (8)	0.8247 (3)	0.4432 (2)	0.0637 (14)	
H10	0.2753	0.8216	0.4841	0.076*	
C11	0.2932 (7)	0.7916 (3)	0.3894 (2)	0.0619 (13)	
H11	0.4268	0.7675	0.3947	0.074*	
C12	−0.2641 (9)	0.9374 (4)	0.4904 (2)	0.105 (2)	
H12A	−0.2614	0.9843	0.4600	0.157*	
H12B	−0.2906	0.9592	0.5328	0.157*	
H12C	−0.3734	0.8978	0.4759	0.157*	
C13	0.7134 (7)	0.6082 (3)	0.2196 (2)	0.0548 (12)	
C14	0.7190 (8)	0.5414 (3)	0.2660 (2)	0.0671 (14)	
H14	0.6117	0.5367	0.2940	0.081*	
C15	0.8826 (9)	0.4826 (3)	0.2703 (2)	0.0787 (16)	
H15	0.8830	0.4387	0.3011	0.094*	
C16	1.0438 (9)	0.4878 (3)	0.2299 (3)	0.0758 (16)	
H16	1.1541	0.4486	0.2335	0.091*	
C17	1.0390 (9)	0.5529 (4)	0.1837 (3)	0.0803 (16)	
H17	1.1473	0.5573	0.1560	0.096*	
C18	0.8745 (8)	0.6116 (3)	0.1782 (2)	0.0685 (14)	
H18	0.8727	0.6539	0.1462	0.082*	
C19	0.2008 (8)	0.8072 (3)	0.0924 (2)	0.0543 (12)	
C20	0.0029 (8)	0.8437 (3)	0.0891 (2)	0.0613 (13)	
H20	−0.0715	0.8438	0.1264	0.074*	
C21	−0.0899 (7)	0.8807 (3)	0.0316 (2)	0.0645 (13)	
H21	−0.2233	0.9050	0.0307	0.077*	
C22	0.0210 (8)	0.8803 (3)	−0.0238 (2)	0.0605 (13)	
C23	0.2184 (8)	0.8437 (3)	−0.0216 (2)	0.0661 (14)	
H23	0.2922	0.8432	−0.0590	0.079*	
C24	0.3083 (7)	0.8077 (3)	0.0356 (2)	0.0641 (13)	
H24	0.4419	0.7835	0.0362	0.077*	
C25	−0.2637 (9)	0.9444 (4)	−0.0914 (2)	0.0928 (18)	
H25A	−0.3566	0.8985	−0.0813	0.139*	
H25B	−0.2949	0.9623	−0.1359	0.139*	
H25C	−0.2826	0.9921	−0.0627	0.139*	

### Atomic displacement parameters (Å^2^)

**Table:**

	*U*^11^	*U*^22^	*U*^33^	*U*^12^	*U*^13^	*U*^23^
N1	0.071 (3)	0.063 (3)	0.045 (2)	0.004 (2)	0.001 (2)	0.001 (2)
O1	0.098 (3)	0.090 (3)	0.056 (2)	0.012 (2)	0.022 (2)	−0.0092 (19)
O2	0.093 (3)	0.089 (3)	0.045 (2)	0.004 (2)	−0.0074 (18)	0.0110 (17)
C1	0.068 (3)	0.055 (3)	0.039 (3)	0.007 (3)	0.005 (2)	0.001 (2)
C2	0.076 (4)	0.063 (3)	0.042 (3)	0.011 (3)	0.001 (2)	0.002 (2)
C3	0.068 (3)	0.058 (3)	0.044 (3)	0.002 (3)	0.001 (2)	0.000 (2)
C4	0.074 (4)	0.065 (3)	0.042 (3)	0.007 (3)	0.008 (3)	−0.002 (2)
C5	0.065 (3)	0.058 (3)	0.042 (3)	0.002 (3)	0.003 (2)	0.000 (2)
C6	0.059 (3)	0.059 (3)	0.044 (3)	0.009 (3)	0.000 (2)	0.002 (2)
C7	0.076 (4)	0.098 (4)	0.042 (3)	0.010 (3)	0.001 (3)	−0.002 (3)
C8	0.068 (4)	0.101 (4)	0.058 (3)	0.022 (3)	0.000 (3)	−0.002 (3)
C9	0.069 (4)	0.072 (4)	0.045 (3)	0.002 (3)	0.013 (3)	−0.001 (3)
C10	0.077 (4)	0.076 (4)	0.037 (3)	0.007 (3)	0.001 (3)	−0.001 (2)
C11	0.069 (3)	0.066 (3)	0.049 (3)	0.013 (3)	−0.002 (3)	0.003 (3)
C12	0.092 (5)	0.138 (5)	0.089 (4)	0.011 (4)	0.033 (4)	−0.029 (4)
C13	0.063 (3)	0.054 (3)	0.046 (3)	0.004 (3)	−0.002 (2)	−0.008 (2)
C14	0.072 (4)	0.072 (4)	0.057 (3)	0.010 (3)	0.006 (3)	0.006 (3)
C15	0.092 (5)	0.073 (4)	0.070 (4)	0.008 (4)	−0.007 (4)	0.005 (3)
C16	0.082 (4)	0.066 (4)	0.077 (4)	0.015 (3)	−0.011 (3)	−0.016 (3)
C17	0.076 (4)	0.091 (4)	0.074 (4)	0.004 (3)	0.007 (3)	−0.015 (3)
C18	0.074 (4)	0.068 (4)	0.064 (3)	0.008 (3)	0.010 (3)	0.002 (3)
C19	0.064 (3)	0.059 (3)	0.040 (3)	0.001 (2)	0.003 (2)	0.000 (2)
C20	0.068 (4)	0.076 (4)	0.041 (3)	0.000 (3)	0.011 (2)	0.004 (2)
C21	0.069 (3)	0.076 (4)	0.047 (3)	0.009 (3)	−0.004 (3)	0.004 (3)
C22	0.078 (4)	0.065 (3)	0.037 (3)	−0.002 (3)	−0.002 (3)	0.001 (2)
C23	0.073 (4)	0.084 (4)	0.041 (3)	0.007 (3)	0.006 (3)	−0.002 (3)
C24	0.065 (3)	0.077 (4)	0.049 (3)	0.006 (3)	−0.003 (3)	0.003 (3)
C25	0.089 (5)	0.114 (5)	0.070 (4)	0.009 (4)	−0.024 (3)	0.015 (3)

### Geometric parameters (Å, °)

**Table:**

N1—C5	1.356 (5)	C12—H12B	0.9600
N1—C1	1.376 (5)	C12—H12C	0.9600
O1—C9	1.379 (5)	C13—C18	1.381 (6)
O1—C12	1.423 (5)	C13—C14	1.406 (6)
O2—C22	1.378 (5)	C14—C15	1.385 (6)
O2—C25	1.430 (5)	C14—H14	0.9300
C1—C2	1.378 (5)	C15—C16	1.370 (6)
C1—C6	1.491 (5)	C15—H15	0.9300
C2—C3	1.402 (5)	C16—C17	1.384 (6)
C2—H2	0.9300	C16—H16	0.9300
C3—C4	1.406 (5)	C17—C18	1.388 (6)
C3—C13	1.483 (6)	C17—H17	0.9300
C4—C5	1.387 (5)	C18—H18	0.9300
C4—H4	0.9300	C19—C20	1.381 (6)
C5—C19	1.512 (6)	C19—C24	1.394 (5)
C6—C7	1.373 (6)	C20—C21	1.403 (5)
C6—C11	1.412 (5)	C20—H20	0.9300
C7—C8	1.404 (6)	C21—C22	1.382 (6)
C7—H7	0.9300	C21—H21	0.9300
C8—C9	1.396 (5)	C22—C23	1.379 (6)
C8—H8	0.9300	C23—C24	1.385 (5)
C9—C10	1.369 (6)	C23—H23	0.9300
C10—C11	1.383 (5)	C24—H24	0.9300
C10—H10	0.9300	C25—H25A	0.9600
C11—H11	0.9300	C25—H25B	0.9600
C12—H12A	0.9600	C25—H25C	0.9600
			
C5—N1—C1	117.0 (4)	C18—C13—C14	117.4 (4)
C9—O1—C12	119.0 (4)	C18—C13—C3	121.8 (4)
C22—O2—C25	118.8 (4)	C14—C13—C3	120.7 (4)
N1—C1—C2	121.9 (4)	C15—C14—C13	120.7 (5)
N1—C1—C6	114.5 (4)	C15—C14—H14	119.6
C2—C1—C6	123.5 (4)	C13—C14—H14	119.6
C1—C2—C3	121.9 (4)	C16—C15—C14	121.2 (5)
C1—C2—H2	119.1	C16—C15—H15	119.4
C3—C2—H2	119.1	C14—C15—H15	119.4
C2—C3—C4	115.3 (4)	C15—C16—C17	118.5 (5)
C2—C3—C13	122.2 (4)	C15—C16—H16	120.7
C4—C3—C13	122.4 (4)	C17—C16—H16	120.7
C5—C4—C3	121.0 (4)	C16—C17—C18	120.9 (5)
C5—C4—H4	119.5	C16—C17—H17	119.6
C3—C4—H4	119.5	C18—C17—H17	119.6
N1—C5—C4	122.8 (4)	C13—C18—C17	121.2 (5)
N1—C5—C19	114.4 (4)	C13—C18—H18	119.4
C4—C5—C19	122.8 (4)	C17—C18—H18	119.4
C7—C6—C11	116.3 (4)	C20—C19—C24	117.5 (4)
C7—C6—C1	122.4 (4)	C20—C19—C5	121.0 (4)
C11—C6—C1	121.2 (4)	C24—C19—C5	121.5 (4)
C6—C7—C8	123.3 (4)	C19—C20—C21	122.4 (4)
C6—C7—H7	118.3	C19—C20—H20	118.8
C8—C7—H7	118.3	C21—C20—H20	118.8
C9—C8—C7	118.5 (4)	C22—C21—C20	118.6 (5)
C9—C8—H8	120.7	C22—C21—H21	120.7
C7—C8—H8	120.7	C20—C21—H21	120.7
C10—C9—O1	116.7 (4)	O2—C22—C23	115.7 (4)
C10—C9—C8	119.3 (4)	O2—C22—C21	124.5 (5)
O1—C9—C8	124.1 (5)	C23—C22—C21	119.8 (4)
C9—C10—C11	121.4 (4)	C22—C23—C24	120.9 (4)
C9—C10—H10	119.3	C22—C23—H23	119.5
C11—C10—H10	119.3	C24—C23—H23	119.5
C10—C11—C6	121.1 (4)	C23—C24—C19	120.7 (4)
C10—C11—H11	119.4	C23—C24—H24	119.6
C6—C11—H11	119.4	C19—C24—H24	119.6
O1—C12—H12A	109.5	O2—C25—H25A	109.5
O1—C12—H12B	109.5	O2—C25—H25B	109.5
H12A—C12—H12B	109.5	H25A—C25—H25B	109.5
O1—C12—H12C	109.5	O2—C25—H25C	109.5
H12A—C12—H12C	109.5	H25A—C25—H25C	109.5
H12B—C12—H12C	109.5	H25B—C25—H25C	109.5

### Hydrogen-bond geometry (Å, °)

**Table:**

*D*—H···*A*	*D*—H	H···*A*	*D*···*A*	*D*—H···*A*
C25—H25C···Cg1^i^	0.96	2.82	3.605	140

Symmetry codes: (i) −*x*, −*y*, −*z*.
